# Pompe disease- experience from South India

**DOI:** 10.1186/1471-2474-14-S2-P23

**Published:** 2013-05-29

**Authors:** Lokesh Lingappa, Radha Rama Devi, Alpa Dherai, Catherene Rehder, Priya Kishnani

**Affiliations:** 1 Deparment of Pediatric Neurology, Rainbow Children’s Hospital and Perinatal Centre, India; 2 Department of Metobolic Medicine and Genetics, Rainbow Children’s Hospital and Perinatal Centre, India; 3 Sandor Proteomics, India; 4 Department of Cytogenetics, Duke University Medical Center, Durham, North Carolina, USA; 5 Department of Pediatrics, Duke University Medical Center, Durham, North Carolina, USA

## Introduction

Herein, we describe our experience with six Pompe patients in South India from September 2007 to July 2012. All patients were diagnosed at our centre by clinical exam and confirmed with enzyme assays and DNA analyses.

## Results

A total of six patients (4 months to 12 months, mean 7.33 months) were diagnosed during the study period. Three presented to cardiologists for breathlessness and infiltrative hypertrophic cardiomyopathy. All had motor delay with severe hypotonia and head lag, respiratory distress, and transaminitis. Three other children presented with hypotonia and motor delay, and one of these was diagnosed during an episode of pneumonia. Novel mutations were found in some of these children. Four of six were born to consanguineous parents, and two of six were able to receive ERT. Through the INCAP programme on a compassionate basis, our patient was the first to receive ERT for Pompe disease in India. The first child started therapy at 8 months of age, and the second at 15 months. Initial improvements were noted in both; however, the first child stopped treatment and died 2 months later at 14 months due to respiratory failure. The second child received 10 infusions and died from progressive respiratory failure at 2 years of age. The other three children died within 2 months of the diagnoses, and one died at 12 months. In two families, we performed antenatal diagnoses using enzyme estimation via amniocentesis and the next pregnancies were not affected.

## Conclusion

Enzyme replacement therapy started at the later ages of 8 and 15 months, respectively, was not effective in infantile onset Pompe disease. The non-availability of governmental support has made ERT virtually out of reach for Indian Pompe patients. Antenatal diagnosis is, however, effective at reducing the disease burden in this highly consanguineous population.

